# Moving global health forward in academic institutions

**DOI:** 10.7189/jogh.06.010409

**Published:** 2016-06

**Authors:** Didier Wernli, Marcel Tanner, Ilona Kickbusch, Gérard Escher, Fred Paccaud, Antoine Flahault

**Affiliations:** 1 Global Studies Institute, University of Geneva, Switzerland; 2 Swiss Tropical & Public Health Institute, Basel, Switzerland; 3 University of Basel, Switzerland; 4 Global Health Programme, Graduate Institute of International and Development Studies, Geneva, Switzerland; 5 Swiss Federal Institute of Technology (EPFL), Lausanne, Switzerland; 6 Institute of Social and Preventive Medicine, Lausanne, Switzerland; 7 Institute of Global Health, Faculty of Medicine, University of Geneva, Switzerland

Global health has attracted growing attention from academic institutions. Its emergence corresponds to the increasing interdependence that characterizes our time and provides a new worldview to address health challenges globally. There is still a large potential to better delineate the limits of the field, drawing on a wide perspective across sciences and geographical areas. As an implementation and integration science, academic global health aims primarily to respond to societal needs through research, education, and practice. From five academic institutions closely engaged with international Geneva, we propose here a definition of global health based on six core principles: 1) cross–border/multilevel approach, 2) inter–/trans–disciplinarity, 3) systems thinking, 4) innovation, 5) sustainability, and 6) human rights/equity. This definition aims to reduce the century–old divide between medicine and public health while extending our perspective to other highly relevant fields. Overall, this article provides an intellectual framework to improve health for all in our contemporary world with implications for academic institutions and science policy.

Health and well–being are major challenges for the 21^st^ century. While these key areas of societal development have gained prominence worldwide by receiving more political attention and funding than ever, the expression ‘global health’ has emerged to describe the profound shift in the nature of health within the context of globalization. Becoming ubiquitous, global health has generated increasing interest from academic institutions, which, as places of knowledge innovation, validation, transmission, and application, have a critical role to play in global health education, research, and practice [1,2]. In this article we use the terms ‘academic global health’ (AGH) to focus on the key role of academic institutions including university hospitals in the global health system. As an integration and implementation science [3], the primary goal of AGH is to foster transformative knowledge, which implies both new models of thinking and new types of research. At the operational level, this translates into a process of mutual learning for change and health improvement, through sharing and comparing across systems and cultures, using both qualitative and quantitative methods, validating new evidence internally and externally, and making interdisciplinary and international collaborations a prerequisite. From the viewpoint of five academic institutions closely engaged with Geneva, a leading city in global health and global governance, the present article attempts to reflect on the core principles, definition, and significance of AGH.

## THE NEW CONTEXT FOR HEALTH

AGH integrates the three traditional areas of health care, international health, and public health and reflects global changes in five key dimensions. First, from a predominantly local or national issue, health has become more transnational as the scope and velocity of the transmission of diseases and their determinants have increased, thereby making broad international collaborations and partnerships indispensable. Second, as epitomized by HIV–AIDS, the distinction between curative individual–based medicine and preventive population–based public health has blurred, requiring a rethink of the provision of public health services and health care delivery as a continuum rather than separate entities. Third, the governance of health and social systems has come to include a broad range of actors beyond governments such as charity, civil society, and the private sector, making a continuous assessment of roles and responsibilities of all actors a necessity. Fourth, the biomedical paradigm rooted dominantly in reductionism and biological determinism has failed to provide sustainable solutions for health and well–being, implying the need to develop broader transdisciplinary approaches. Fifth, the interdependence of health with other sectors, together with foreign policy agendas such as trade, security, human rights, environment, and development, has been increasingly recognized, requiring systemic approaches through which diseases and health problems are positioned within broader social, ecological and political systems. Clearly, contemporary global changes have decreased the capacity of the 20^th^ century dominant conceptualizations of international health, and to a lesser extent health care and public health, to address current health challenges effectively. As AGH emanates from this new context for health, we propose six core principles to guide global health research, education, and practice.

## SIX CORE PRINCIPLES FOR AGH

### AGH addresses cross–border and multi–level health issues

As health issues increasingly cross national boundaries, we need to understand how phenomena occurring at different spatial and temporal scales interface. For example, multilevel geo–ecological frameworks explore how determinants shape health from micro/local to macro/global levels drawing on the progress of scientific knowledge in many fields. Globalization is not a simple process and not everything is global: we constantly face complex “fragmegrative” dynamics where globalizing forces are counteracted by localizing ones [4]. Thus, continuous communication between local communities and academics or professionals working at different levels and diverse geographic areas is crucial to optimize the tailoring of local interventions while avoiding fragmentation of the strategies regionally and globally. A comparable relevant example is the interrelated area of climate change. As addressing the root global causes of climate change is impossible at the local/national level alone, an understanding of scientific evidence facilitated by the UN’s Intergovernmental Panel on Climate Change can direct these constituencies toward well–tailored adaptation and mitigation policies.

### AGH mobilizes all relevant academic disciplines

While traditional academic disciplines identify, delimit, and analyze phenomena, they tend to produce hyper–specialization, which in turn can result in fragmented understanding and actions in silos. Fragmentation is amplified by the enormous amount of knowledge produced within and outside the academy. As philosopher of science Karl Popper put it: “we are not students of some subject matter, but students of problems” [5], meaning that addressing complex societal problems such as the Ebola crisis in West Africa in 2014 transcends the boundaries of academic disciplines. Consequently, AGH should not be conceptualized as a new discipline but rather as a “transdiscipline” that seeks to integrate knowledge from different sources. Although currently AGH is, still mostly multidisciplinary, corresponding to a juxtaposition of disciplinary perspectives, it should become more interdisciplinary integrating insights from all relevant academic disciplines. Even better it should aim to become transdisciplinary, integrating insights from all relevant disciplines and actors outside academia to address problems too complex for a single discipline or sector [6].

### AGH studies complex systems in the real world

Systems science, which encompasses a broad set of theories and methods developed in life sciences, social sciences and engineering during the 20^th^ century, focuses on the principles that govern living and social systems. From cells to global governance, global health refers to complex systems. These systems are constituted of multiple components interacting through reinforcing or inhibiting feedback loops, they operate in constantly evolving contexts, and they typically exhibit properties that result not from specific components of the system but from their interactions, such as nonlinear behavior, self–organization, and emergence [7]. By analyzing the roles, positions, responsibilities and interdependencies of the different building blocks of global health systems, systems science modifies our mental boundaries, generates new questions and hypotheses, and improves our models which can in turn reduce policy failure. While a system perspective is critical to address major health problems in the real world, AGH does not rule out reductionist and selective approaches, as basic reductionist research is a major driver of scientific progress and as selective approaches has been highly successful in some cases (eg, eradication of smallpox). Overall, AGH aims to provide an integrated intellectual framework for debating, experimenting, and implementing options.

### AGH seeks to provide affordable, effective, and integrated innovation

The exponential growth of scientific and technological knowledge is key to improving health and well–being both in high–income countries (HIC) and low– and middle–income countries (LMIC). Technologies for global health include both health technologies (ie, vaccines, e–health, genomics) and technologies that “*have health benefits that arise from use outside of health, such as the Internet or irrigation”* considering that “*most health problems are best addressed by a combination of technologies*” [8]. In addition global health relies strongly on computer technologies to use and model the increasing amount of data (data science) for example in worldwide disease surveillance. Innovation in global health also takes place at the social and policy levels [9]. Social and policy innovation for health encompasses all strategies to improve the uptake of technological innovation, to promote health and well–being, and to address broader problems such as access to education. While in the 20^th^ century health innovation used to flow exclusively from HIC to LMIC, HIC can also benefit from innovation in LMIC (reverse innovation) including from the social innovation capacity of communities, and promotes mutual learning for change.

### AGH is concerned with sustainability

With rapid population growth combined with unsustainable modes of production and consumption, ever growing constraints apply on the planet. In this early 21^st^ century, humanity is facing the huge challenge to learn to live within planetary boundaries [10]. From anthropocentric models of socio–economic development, we need to include the environmental dimension into the equation and shift to sustainable development. Sustainability science, defined as the study of “*the interactions between natural and social systems, and with how those interactions affect the challenge of sustainability: meeting the needs of present and future generations while substantially reducing poverty and conserving the planet’s life support systems*” [11] is an integral part of global health. Health is a prerequisite–good health and well–being are required for people to achieve their full potential–and an outcome of sustainable development. As the health and fate of humanity ultimately depends on Earth’s natural systems, AGH is thus essential to shape sustainable development goals, to measure progress toward human well–being, and to improve our understanding of how environmental, social, economic, and health goals can be integrated to preserve planetary health [10,12].

### AGH is committed to the normative framework of human rights and equity

Health is an essential part of the broad normative framework of human rights and social justice as affirmed by the World Health Organization preamble. Indeed, several international treaties consider health a human right, which imposes obligations on states to respect, to protect, and to contribute to its progressive realization. Beyond access to health care, the right to health covers social determinants of health, since living conditions are broadly shaped by the distribution of resources and power [13], the rule of law, and levels of liberty, security, and dignity. Central to AGH is understanding the distribution and impact of the unfair and avoidable differences (inequities) in health status between population, genders, and countries, and the reduction of these inequities through action within and beyond the health sector. Universal Health Coverage, the provision of health services with adequate financial protection for all, should thus be enshrined within the broader right to health [14] and the overarching goal of reducing poverty, the main single obstacle to health with 896 million people living with less than a US$ 1.90/day and 2.1 billion below US$ 3.1/day according to the World Bank in 2012 [15].

## DEFINITION AND CHALLENGES FOR ACADEMIC INSTITUTIONS

Based on the six principles above, we propose the following definition of AGH: *Within the normative framework of human rights, global health is a system–based, ecological and transdisciplinary approach to research, education, and practice which seeks to provide innovative, integrated, and sustainable solutions to address complex health problems across national boundaries and improve health for all.* This definition first underlines the dynamic complexity which results from our era of interdependence [16]. Within the progressive differentiation of scientific knowledge, it aims to reconcile the century–old divide between medicine and public health, while extending our perspective to other highly relevant fields such as engineering and international relations. Second, this definition corresponds to the perspective of five Swiss academic institutions closely engaged with international Geneva as the main hub of global health governance. While we believe that it reflects the challenges associated with addressing health issues across the world, we consider our work as a proposal to foster further debate with researchers in other countries especially from the global South. Third, translating this definition into concrete projects regarding education, research, and partnerships is key to move AGH forward. **Table 1** summarizes projects based in our five academic institutions which contribute to the conceptualization of global health presented here.

**Table 1:** Examples of programs in global health based at five Swiss academic institutions

Program name and institution	Short description
Master of Science and PhD in global health, Institute of Global Health and Global Studies Institute, University of Geneva	As innovative educational programs in global health, the PhD is an executive program based on blended learning (residential weeks in Switzerland, highly intensive distance learning, and accredited MOOCs) while the Master is a transdisciplinary two–year full time program based in Geneva with specialization in other training programs.
EssentialTech Initiative, Swiss Federal Institute of Technology in Lausanne (EPFL)	The aim of this cooperation and research initiative is to foster the development and implementation of essential technologies including medical equipment, water, and sanitation, which can contribute to improve health in LMIC.
Long term partnership with Ifakara, Tanzania, Swiss Tropical and Public Health Institute in Basel (SwissTPH)	The SwissTPH has a long–term collaboration with the Ifakara Health Institute (IHI) in Tanzania, a successful institution for basic and translational health research, education and support in public health. While the IHI has been a Tanzanian institution since 1996, the model of building comparable centers has spread through SwissTPH and partners to other countries in Africa.
Research on chronic diseases, Institute of social and preventive medicine (IUMSP) in Lausanne	The IUMSP specializes in research on epidemiology and prevention of chronic diseases, particularly cancers and cardiovascular diseases as the burden of these conditions are growing in aging societies and requires new public health responses.
Executive Training in Global Health Diplomacy, Global Health Programme, Graduate Institute of International and Development Studies in Geneva	Since 2007, the Global Health Programme offers executive training in global health diplomacy around the world with the aim of bringing together diplomats and health decision–makers to understand their common interests in health as a goal of foreign policy.

In education, the main challenge is to extend the topics and methods taught both in the curricula of global health in medicine, public health, and engineering, and in other programs granting global health degrees, while maintaining sufficient coherence and disciplinary depth. Mixing students from diverse backgrounds is paramount to foster collaboration across disciplines and to develop the reflexive and synthesizing mind in a competence–based education. Educational models such as interdisciplinary co–teaching and the introduction of existing textbooks for interdisciplinary teaching in the curricula can help. In addition, advances in e–learning and particularly massive open online courses (MOOCs) can effectively complement curricula. MOOCs offer unprecedented opportunities to create large scale horizontally and vertically integrated learning communities.

**Figure 1:**
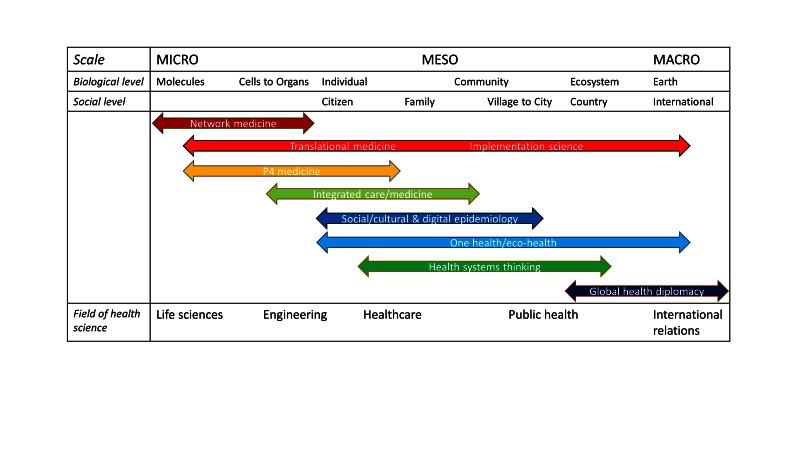
Scope of selected interdisciplinary research and education approaches in academic global health.

In research, this definition requires collaborative or transdisciplinary ‘team science’ with knowledge increasingly produced through teams and networks of scholars. Some key areas of interdisciplinary enquiry are mentioned in **Table 2** while their scope is presented within the wider context of global health in **Figure 1**. As differences of disciplinary cultures and paradigms are common obstacles for interdisciplinary research, dedicated support from academic institutions, funding agencies, and governments can help alleviate these barriers. Leading medical journals already play their part by publishing perspectives from non–medical disciplines although the format for research submissions often still remains too rigid [18]. More importantly, the obstacles associated with an interdisciplinary academic career pathway remain a major issue almost everywhere. Traditional disciplinary candidates are favored when it comes to promotion and tenure for faculty position [19]. As AGH needs to work across academic disciplines, AGH programs may be organized in interfaculty or interdisciplinary centers with joint appointments [20] and/or work as network of actors across institutions and disciplines.

**Table 2:** Selected interdisciplinary research and education approaches relevant to global health

Approaches	Description
Network medicine	Part of network science, network medicine seeks to improve our understanding of disease mechanisms and pathways. It focuses on measuring and analyzing the structures and dynamics of complex molecular networks, which entails relationships between multiple components at the cellular level. Network medicine contributes to better understanding the genetic interlinkages between diseases, and provides insight for new treatments and diagnostics. It also provides a basis to place disease systems into the context of health and social systems.
P4 medicine	Progress and cost reduction in biotechnologies are enabling a more predictive, preventive, personalized, and participatory medicine (P4), which takes into account the genetic background and other specificities of each patient as well as their economic context. The ambition of P4 medicine is to offer customized treatment and improve the detection of diseases before symptoms appear. While medicine has been largely reactive to diseases, P4 is proactively garnering a range of data to maintain well–being.
Translational medicine/Implementation science	Implementation science (IS) is firmly based on evidence from basic science and corresponds to a continuum of knowledge translation activities, which aims to reduce the science to policy and practice gaps. In medicine, translational medicine is the processes of transforming basic science and technologies from bench to bedside and population. In public health, IS plays a key role in validating health interventions seeking to reach all those who need them in order to improve population/community health effectively and equitably.
Integrated care/medicine	Integrated care (IC) seeks to address patient problems in holistic ways rather than only through specialized care to improve health care delivery (eg, quality, satisfaction, access). As a bottom–up person–centered perspective, IC responds to the fragmentation of health care delivery due to progressive hyper–specialization of medicine. An example of integrated care is the development of family medicine where the general practitioners play the role of gatekeeper.
Health and social systems thinking	Health systems are complex open systems with several blocks. Thus, health systems thinking focuses on understanding the roles, functions and positions of the systems’ building blocks as well as the complex positive or negative feedback loops between these blocks [22]. It provides a framework to strengthen health and social systems, for example through integrated locally tailored interventions between vertical programs and primary health care.
One Health/eco–health	A “One Health” approach seeks to address, in an integrated way, health issues that result from the interplay of multiple human, animal, and environmental factors within a given socio–ecological context. This approach is timely as zoonoses are the main source of emerging and re–emerging infectious diseases (eg, bird flu, SARS, HIV or Ebola) due to several factors such as the ever increasing mobility of human population, disruptions of ecosystems, industrialization of food systems, and socio–political fragility.
Social/cultural and digital epidemiology	While social/cultural epidemiology mixes epidemiology with social theories, digital epidemiology uses a broad range of digital data sources and computer science. Social/cultural epidemiology establishes causal relationships between economic, social and political conditions in which people live as well as health status over their life–course. Digital epidemiology not only provides information about outbreaks and diseases dynamics but also examines and predicts how health and diseases are spread through social ties and networks.
Global health diplomacy	Global health diplomacy (GHD) is concerned with understanding how we collectively deal with cross–border health issues and global challenges through bilateral or multilateral negotiations across different countries, actors, levels and systems. GHD sheds light on the political nature of health, the competing social norms, the evolving role of myriad actors and the complex scientific and political processes that surround any health issue.

Currently AGH attracts more attention in HIC than in LMIC, reflecting a wider gap in research and education capacities. While the concept of global health originated and diffused widely in HIC, the long term relevance and success of AGH depends on its use and appropriation by academic institutions in LMIC. Three components are essential in this regard. First, international collaboration is critical for both teaching/learning and research in global health but should not “be a one–way street” [21] and should benefit all partners in HIC and LMIC. One challenge is to depart from a long (neo–) colonialist tradition associated with international and tropical medicine. The development of ethical guidelines for educational exchange is a step in the right direction [22]. In addition, AGH requires more South–South collaboration under the leadership of countries such as China or Brazil whose size are critical for capacity building and outreach. Finally, there is a role to play for international academic bodies such as the World Federation of Academic Institutions for Global Health in promoting an inclusive vision of global health and in reflecting on the future of the field based on a broad geographic representation of academic institutions.

## CONCLUSION

Within the knowledge society one of the most important challenges faced by academic institutions is to keep their societal relevance. One way forward is to create and develop new intellectual spaces to pursue the production of knowledge across disciplines while drawing on the achievements of two centuries of disciplinary organization of science. As an integration and implementation science, AGH offers such a space to advance our understanding of complex problems and comply with the social responsibility of academic institutions to contribute to societal well–being and sustainable development.
